# Anaerobic Conversion of Saline Phenol-Containing Wastewater Under Thermophilic Conditions in a Membrane Bioreactor

**DOI:** 10.3389/fbioe.2020.565311

**Published:** 2020-09-30

**Authors:** Julian D. Muñoz Sierra, Víctor S. García Rea, Daniel Cerqueda-García, Henri Spanjers, Jules B. van Lier

**Affiliations:** ^1^Section Sanitary Engineering, Department of Water Management, Delft University of Technology, Delft, Netherlands; ^2^KWR Water Research Institute, Nieuwegein, Netherlands; ^3^Institute of Ecology, National Autonomous University of Mexico, Mexico City, Mexico

**Keywords:** thermophilic, high salinity, AnMBR, phenol, microbial community, hydrogenotrophic activity

## Abstract

Closing water loops in chemical industries result in hot and highly saline residual streams, often characterized by high strength and the presence of refractory or toxic compounds. These streams are attractive for anaerobic technologies, provided the chemical compounds are biodegradable. However, under such harsh conditions, effective biomass immobilization is difficult, limiting the use of the commonly applied sludge bed reactors. In this study, we assessed the long-term phenol conversion capacity of a lab-scale anaerobic membrane bioreactor (AnMBR) operated at 55°C, and high salinity (18 gNa^+.^L^–1^). Over 388 days, bioreactor performance and microbial community dynamics were monitored using specific methanogenic activity (SMA) assays, phenol conversion rate assays, volatile fatty acids permeate characterization and Illumina MiSeq analysis of 16S rRNA gene sequences. Phenol accumulation to concentrations exceeding 600 mgPh^.^L^–1^ in the reactor significantly reduced methanogenesis at different phases of operation, while applying a phenol volumetric loading rate of 0.12 gPh^.^L^–1.^d^–1^. Stable AnMBR reactor performance could be attained by applying a sludge phenol loading rate of about 20 mgPh^.^gVSS^–1.^d^–1^. *In situ* maximum phenol conversion rates of 21.3 mgPh^.^gVSS^–1^^.^d^–1^ were achieved, whereas conversion rates of 32.8 mgPh^.^gVSS^–1^^.^d^–1^ were assessed in *ex situ* batch tests at the end of the operation. The absence of caproate as intermediate inferred that the phenol conversion pathway likely occurred via carboxylation to benzoate. Strikingly, the hydrogenotrophic SMA of 0.34 gCOD-CH_4_^.^gVSS^–1^^.^d^–1^ of the AnMBR biomass significantly exceeded the acetotrophic SMA, which only reached 0.15 gCOD-CH_4_^.^gVSS^–1^^.^d^–1^. Our results indicated that during the course of the experiment, acetate conversion gradually changed from acetoclastic methanogenesis to acetate oxidation coupled to hydrogenotrophic methanogenesis. Correspondingly, hydrogenotrophic methanogens of the class Methanomicrobia, together with Synergistia, Thermotogae, and Clostridia classes, dominated the microbial community and were enriched during the three phases of operation, while the aceticlastic Methanosaeta species remarkably decreased. Our findings clearly showed that highly saline phenolic wastewaters could be satisfactorily treated in a thermophilic AnMBR and that the specific phenol conversion capacity was limiting the treatment process. The possibility of efficient chemical wastewater treatment under the challenging studied conditions would represent a major breakthrough for the widespread application of AnMBR technology.

## Introduction

Phenols are major contaminants found in wastewaters of several chemical industries, which are often discharged at high temperatures (Busca et al., 2008; Rosenkranz et al., 2013; Wang et al., 2017). Additionally, closing water loops in the chemical sector often result in concentrated, highly saline wastewaters, which increases the complexity of the produced wastewater (Muñoz Sierra et al., 2017). Despite the existing physicochemical processes applied for phenol removal, i.e., membrane distillation, pervaporation, adsorption, extraction, nanofiltration, reverse osmosis, and oxidation processes (wet air, electrochemical, ozonation, UV/H_2_O_2_, Fenton) (Villegas et al., 2016; Raza et al., 2019); biological treatment processes are preferred due to its cost-effectiveness. In this regard, anaerobic treatment offers the advantages of minimal energy requirement, low sludge production, and the conversion of organic pollutants into energy-rich biogas. Under both saline and high-temperature conditions, effective biomass immobilization becomes cumbersome, constraining the application of anaerobic sludge bed systems to treat these wastewaters (Dereli et al., 2012; van Lier et al., 2015). Moreover, the phenol degrading capacity of methanogenic consortia is generally very low and is expected to develop only slowly. Therefore, combining anaerobic treatment with membrane assisted biomass separation is an attractive option when phenol conversion is required at high salinity, and thermophilic conditions (Lin et al., 2013; Muñoz Sierra et al., 2018b).

When chemical wastewaters are at high temperatures, direct thermophilic treatment becomes of interest because it reduces the need for cooling the wastewater. Particularly when process water reclamation is envisaged, maintaining the temperature reduces the overall energy requirement. Despite its potentials (van Lier, 2008), thus far, full-scale anaerobic membrane bioreactors (AnMBRs) are not applied for high-temperature chemical wastewater treatment (Duncan et al., 2017). Only a few previous studies have shown the potential of thermophilic conditions for treating phenolic compounds in continuous reactors (Wang et al., 2011; Ramakrishnan and Surampalli, 2013). Wang et al. (2011) compared UASB reactors under mesophilic and thermophilic conditions and concluded that thermophilic anaerobic digestion improves about 30% the degradation of phenolic compounds. Likewise, Ramakrishnan and Surampalli (2013) suggested that thermophilic conversion of phenolic wastewater in an anaerobic hybrid reactor is superior to mesophilic in terms of methane yield, effluent quality, and stability.

Conversely, other studies have also found drawbacks of treating phenol containing wastewater under thermophilic conditions. Fang et al. (2006) indicated that the phenol conversion rate at 55°C in a UASB reactor was significantly lower than under mesophilic conditions. Furthermore, Levén and Schnürer (2005) concluded that phenolics are mineralized to methane and carbon dioxide under mesophilic conditions, whereas under thermophilic conditions, only benzoic acid is degraded. Muñoz Sierra et al. (2018b) suggested that under mesophilic and hyper-mesophilic conditions (42–45°C), the phenol conversion capacity of an AnMBR at high salinity is more stable compared to thermophilic conditions. However, because the operation at 55°C in that study was carried out only during a short-term, it remains unclear whether or not a stable phenol degrading methanogenic consortium may develop. Therefore, this study aims to determine the maximum conversion capacity of a laboratory-scale AnMBR during a long-term operation at 55°C, treating phenol-containing wastewater at 18 gNa^+.^L^–1^_._ Moreover, the microbial community activity and structure in response to increasing phenol loading rates along with three phases of operation were evaluated.

## Materials and Methods

### Experimental Set-Up and Operation

**FIGURE 1:**
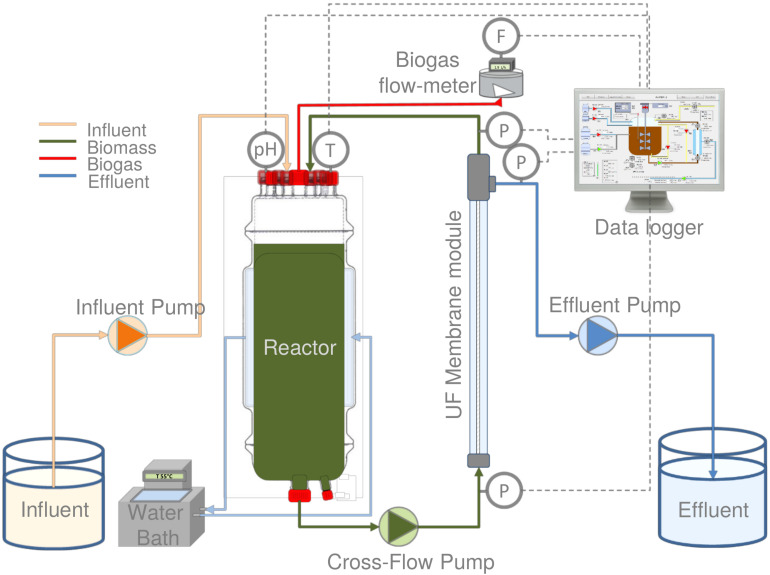
Schematic representation of the thermophilic AnMBR.

The experiments were performed by using a 6.5 L laboratory-scale AnMBR reactor, equipped with an ultra-filtration (UF) side-stream membrane module (Figure 1). A tubular polyvinylidene fluoride membrane (X-flow, Pentair, Netherlands) with 5.2 mm inner diameter, 0.64 m length and 30 nm nominal pore size was used. The reactor was equipped with feed, recycle, and effluent pumps with 4–20 mA variable speed (120U/DV, 220Du, Watson-Marlow, Netherlands), pH, and temperature sensors (Memosens, Endress & Hauser, Germany), and a biogas meter (Milligas Counter MGC-1 PMMA, Ritter, Germany). Transmembrane pressure (TMP) was measured by using three pressure sensors (AE Sensors ATM, Netherlands). The temperature of the jacketed reactor was controlled under thermophilic conditions by a thermostatic water bath (Tamson Instruments, Netherlands). The entire set-up was controlled by a programmable logic controller (PLC) connected to a PC with LabVIEW software (version 15.0.1f1, National Instruments, United States).

The AnMBR was operated at 55.0 ± 0.8°C for 388 days. During this time, the reactor was fed with synthetic phenolic wastewater with phenol concentration between 50 and 800 mgPh^.^L^–1^ and a sodium concentration of 18 gNa^+.^L^–1^. The experiment was divided into three phases (I, II, and III). In all of the phases, the phenol concentration was increased step-wise to determine the maximum conversion capacity and the attainable phenol loading rate of the AnMBR. The applied total organic loading rates (OLRs) were between 2.0 and 4.0 gCOD^.^L^–1.^d^–1^ during all phases (Table 1). The OLRs were calculated as OLR = influent COD (g.L^–1^) ^∗^ Flow rate (L.d^–1^)/AnMBR volume (L). The applied flow rate was 1.0 L.d^–1^. An average solids retention time (SRT) of about 120 ± 13 days was maintained. The average SRT was calculated periodically as SRT_*av*_ = X (g VSS in the AnMBR)_*av*_/X removed_*net*_ (gVSS.d^–1^), where X removed_*net*_ resulted from the biomass removed.day^–1^ (sampling for tests) and the biomass returned.day^–1^ (after some tests).

**TABLE 1:** Operational organic and phenol loading rates of the thermophilic AnMBR.

Phase	Days	OLR [gCOD⋅L^–1^⋅d^–1^]	Phenol loading rate [gPh⋅L^–1^⋅d^–1^]	Phenol influent concentration [g⋅L^–1^]
I	0–12	3.7	0.01	0.05
	13–42	3.7	0.02	0.1
	43–69	3.8	0.05	0.3
	70–96	3.9	0.09	0.6
	97–108	4.0	0.12	0.8
	109–133	2.3	0.12	0.8
II	134–155	2.0	0.02	0.1
	156–198	2.1	0.03	0.2
	199–219	2.2	0.08	0.5
	220–241	2.3	0.11	0.7
	242–262	2.3	0.12	0.8
III	263–325	2.0	0.01	0.08
	326–332	2.1	0.02	0.15
	333–349	2.1	0.04	0.25
	350–388	2.1	0.03	0.2

The AnMBR was completely mixed applying a turnover of 170 times.d^–1^. The membrane unit was operated at a cross-flow velocity of 0.65 m s^–1^. A cyclic membrane filtration operation was carried out, consisting of 500 s filtration and 30 s backwash. Backwash was done by reversing the permeate pump flow. An operational flux of 4.0 L^.^m^–2.^h^–1^ was applied as a result of the experimental settings. The permeate flow was controlled with the variable speed of the effluent and influent and pumps, and it was regularly double-checked manually. An average TMP of 177 ± 92 mbar and a total membrane filtration resistance of 9.77 × 10^12^ [1/m] could be maintained during the AnMBR long-term operation.

### Inoculum and Wastewater Composition

The reactor was initially inoculated with mesophilic anaerobic biomass obtained from a full-scale UASB reactor at 8 gNa^+^^.^L^–1^ (Shell, Moerdijk, Netherlands) and subjected to hyper-mesophilic and thermophilic conditions at 16 gNa^+^^.^L^–1^ before the start of the experiment. The synthetic phenolic wastewater consisted of phenol (0.1–0.8 gPh^.^L^–1^), acetate (10–20 g^.^L^–1^), yeast extract (2.0 g^.^L^–1^), NaCl, K_2_HPO_4_, KH_2_PO_4_, varying according to the applied organic and phenol loading rates (Table 1), while maintaining 18 gNa^+^^.^L^–1^ and a K^+^:Na^+^ ratio of 0.05. Macronutrients (9 mL^.^L^–1^), and micronutrients (4.5 mL^.^L^–1^) solutions were added. Macronutrients solution contained (in g^.^L^–1^): NH_4_Cl (170), CaCl_2_.2H_2_O (8), and MgSO_4_.7H_2_O (9); micronutrients solution contained (in g L^–1^): FeCl_3_.6H_2_O (2), CoCl_2_.6H_2_O (2), MnCl_2_.4H_2_O (0.5), CuCl_2_.2H_2_O (0.03), ZnCl_2_ (0.05), H_3_BO_3_ (0.05), (NH_4_)_6_Mo_7_O_2_.4H_2_O (0.09), Na_2_SeO_3_ (0.1), NiCl_2_.6H_2_O (0.05), EDTA (1), Na_2_WO_4_ (0.08). The chemical reagents were of analytical grade.

### Volatile Fatty Acids

Prior analysis, 10 mL of the AnMBR permeate samples was filtrated over 0.45 μm filter paper. The filtrated liquid was diluted with pentanol (300 mg.L^–1^). 10 μL of formic acid (purity > 99%) was added into the final 1.5 mL vials. Volatile fatty acids (VFAs) were measured by gas chromatography (GC) using an Agilent 19091F-112, 25 m × 320 μm × 0.5 μm column and an FID detector (Agilent 7890A, United States). The sample injection volume was 1 μL. Helium was used as carrier gas with a total flow rate of 67 mL/min and a split ratio of 25:1. The GC oven temperature was programmed to increase from 80 to 180°C in 10.5 min. The temperatures of the injector and detector were 80 and 240°C, respectively.

### Permeate Characterization

Phenol concentrations were measured using high-performance liquid chromatography HPLC LC-20AT (Shimadzu, Japan) equipped with a 4.6 mm reversed-phase C18 column (Phenomenex, Netherlands) and a UV detector at a wavelength of 280 nm. The mobile phase used was 25% (v/v) acetonitrile at a flow rate of 0.95 mL^.^min^–1^. The column oven was set at 30°C. Fast phenol measurements were also carried out by Merck – Spectroquant Phenol cell kits by using a spectrophotometer NOVA60 (Merck, Germany). Hach Lange kits were used to measure chemical oxygen demand (COD). The COD was measured using a VIS – spectrophotometer (DR3900, Hach Lange, Germany) making proper dilutions to minimize interference by high chloride concentrations, without compromising the accuracy of the measurement.

### Anaerobic Phenol Conversion Rates

Batch tests were conducted in triplicate at the end of phase II to assess the phenol conversion recovery after AnMBR performance perturbation. The volatile suspended solid (VSS) were analyzed according to standard protocols using the lowest possible sample volume (APHA, 2005). Biomass samples were taken with a 150 mL syringe and transferred to 500 mL Schott glass bottles. The bottles were filled to a volume of 400 mL with AnMBR biomass (0.68 g VSS), and a medium containing acetate (3.1–4.6 g^.^L^–1^), phenol (60–109 mgPh^.^L^–1^), 6 mL^.^L^–1^ macro- and 0.6 mL^.^L^–1^ micronutrients solution, 10 mM phosphate buffer solution, and 18 gNa^+.^L^–1^. Three consecutive feedings of the medium were applied. Initial COD and phenol concentrations varied between 3.4–5.1 gCOD^.^L^–1^ and 60–109 mgPh^.^L^–1^, respectively. Temperature and mixing were controlled in an orbital incubator shaker (New Brunswick Biological Shakers Innova 44/44R, United States) at 55°C and 120 rpm respectively. Periodically, liquid samples were taken, and phenol and COD concentrations were measured. Phenol conversion rates [mgPh^.^gVSS^–1^^.^d^–1^] were calculated by using the slope of the phenol concentration vs time curve in each bottle. After the batch tests were finished, the supernatant was removed and biomass was returned to the AnMBR. Similarly, at the end of the AnMBR operation at day 388, biomass samples were taken, and phenol conversion batch tests were carried out with initial phenol concentrations of 40 and 60 mgPh^.^L^–1^.

### Specific Acetotrophic and Hydrogenotrophic Methanogenic Activity

Specific acetotrophic methanogenic activity (SMA) tests were performed in triplicate using an automated methane potential test system (AMPTS, Bioprocess Control, Sweden). All the SMA tests were carried out at 55°C, following the method described by Spanjers and Vanrolleghem (2016).

For the hydrogenotrophic methanogenic activity, 250 mL Schott glass bottles were filled with biomass (0.57 g VSS) and medium (6 and 0.6 mL^.^L^–1^ macro- and micro-nutrients solution, respectively and 10 mM phosphate buffer solution at pH 7.0) to a liquid volume of 200 mL. The gas-phase of the bottles was exchanged by using a gas exchange board (G.R Instruments B.V, Netherlands) with a gas mixture of 80% CO_2_ and 20% H_2_ to an end pressure of 0.5 bar during 5 continuous automated cycles to ensure the complete absence of oxygen. Bottles were incubated in an orbital shaker (New Brunswick Biological Shakers Innova 44/44R, United States) at 55°C and 120 rpm for 10 days, and biogas production was calculated using the exact headspace volume and the drop in headspace pressure versus time. The headspace pressure was measured as described by Coates et al. (1996) using a pressure transducer. The methane content of the biogas was analyzed by using a gas chromatograph 7890A (GC) (Agilent Technologies, United States) equipped with a front thermal conductivity detector (TCD). The temperature of the oven was 45°C for 6 min, then 25°C/min to 100°C. The temperatures of the front inlet, and a front detector were both 200°C.

### Microbial Community Analysis

Biomass samples were taken from the AnMBR on days 88, 241, and 376 to evaluate the microbial community. The DNA extraction was performed from 0.5 g of biomass by using the DNeasy UltraClean Microbial Kit (Qiagen, Hilden, Germany). Agarose gel electrophoresis and Qubit3.0 DNA detection (Qubit dsDNA HS Assay Kit, Life Technologies, United States) were used for quality and quantity control of the DNA. The amplification of the 16S rRNA gene (V3–V4 region) was performed and followed by high throughput sequencing using the MiSeq Illumina platform (BaseClear, Leiden, Netherlands) using the primers 341F (5′-CCTACGGGNGGCWGCAG-3′) and 785R (5′-GACTACHVGGGTATCTAATCC-3′). The Illumina fastq reads (2 × 250) were processed in the QIIME2 pipeline (2018.7) (Bolyen et al., 2019). Reads were quality filtered, denoised, and the amplicon sequences variants (ASVs) were resolved with the DADA2 plugin (Callahan et al., 2016), removing chimeras with the “consensus” method. The taxonomic classification of the representative sequences of ASVs was performed with the “classify-consensus-vsearch” plugin (Rognes et al., 2016) using the SILVA (132) database as a reference. The representative sequences were aligned with the MAFFT algorithm (Katoh and Standley, 2013), and a phylogenetic tree was constructed with FastTree (Price et al., 2010). The feature table and tree were exported to the R environment. Differential abundance analyses between reactor operation phases were performed with the DESeq2 library (Love et al., 2014). The abundance and the tree were visualized with the phyloseq library (McMurdie and Holmes, 2013). ASVs with differential abundances within the operational phases were analyzed with BLAST against the refseq RNA database to identify the closest related species. The sequences reported in this paper have been deposited at ENA under the study accession number PRJEB38467.

## Results

### Thermophilic AnMBR Performance

**FIGURE 2:**
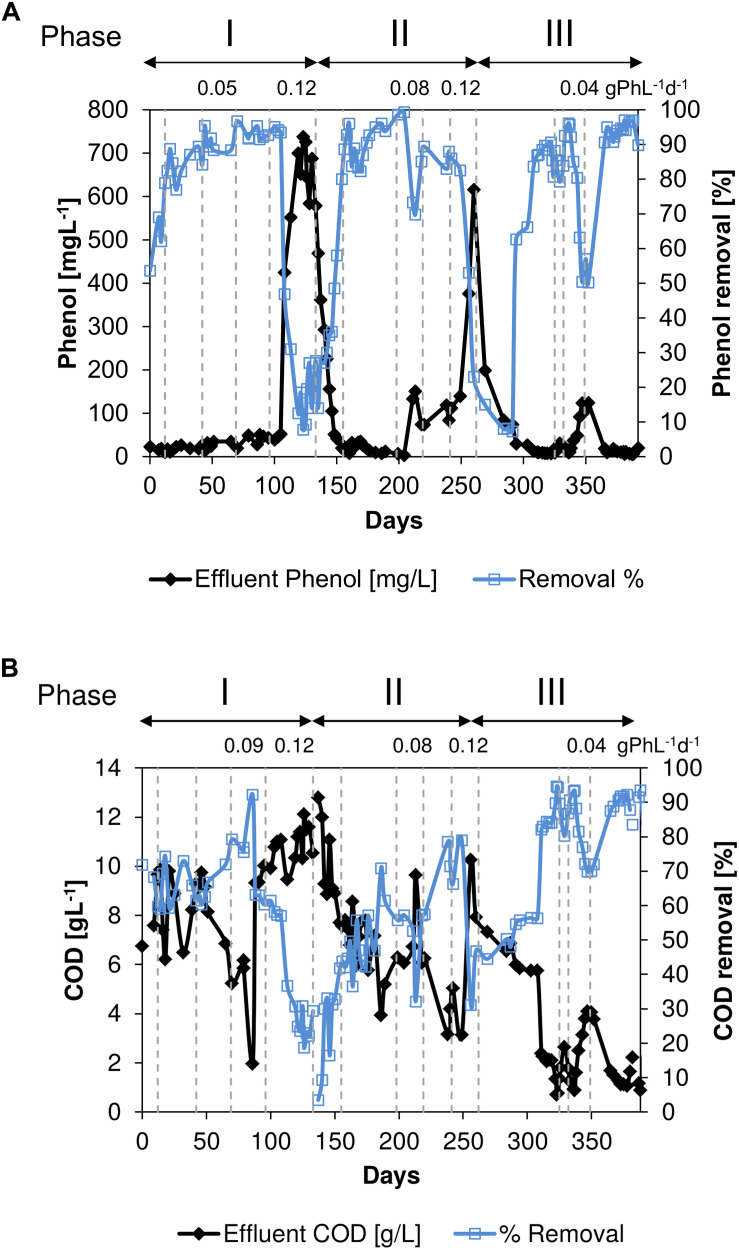
**(A)** Phenol removal efficiency and phenol concentration in the effluent and **(B)**. COD removal efficiency and COD concentration in the effluent during the long-term thermophilic (55°C) condition. The vertical dotted lines indicate the changes in phenol loading rate (gPh⋅L^–1^⋅d^–1^).

Influent and effluent phenol and COD concentrations during the long-term thermophilic AnMBR operation are shown in Figure 2. During days 0–96 in phase I, the effluent COD concentrations were in the range of 2.0–10.0 gCOD^.^L^–1^, and the corresponding removal efficiencies were between 59.0 and 92.3% (Figure 2B) at an average OLR of 3.8 gCOD^.^L^–1.^d^–1^. The increase in the phenol loading rate (Table 1) from 0.01 to 0.09 gPh L^–1^d^–1^ concomitantly increased the phenol removal efficiency from 54 to 95% (Figure 2A). At the end of phase I, the phenol concentration in the reactor reached about 738 mgPh^.^L^–1^ (Figure 2A).

On day 199 in phase II, the phenol loading rate was increased from 0.03 gPh^.^L^–1.^d^–1^ (6.5 mgPh^.^gVSS^–1.^d^–1^) to 0.08 gPh^.^L^–1.^d^–1^ (17.4 mgPh^.^gVSS^–1.^d^–1^) which resulted in an increase in converted phenol from 188 to 349 mgPh^.^L^–1^. Concomitantly, the phenol removal efficiency dropped to 24% (Figure 2A) and the COD removal efficiency to 32% (Figure 2B). However, on day 240 in phase II, the phenol removal efficiency increased to 88%, while applying a phenol loading rate of 0.11 gPh^.^L^–1.^d^–1^ (18.4 mgPh^.^gVSS^–1.^d^–1^). When the phenol loading rate was further increased to 0.12 gPh^.^L^–1.^d^–1^ (20.1 mgPh^.^gVSS^–1.^d^–1^) the reactor performance again deteriorated, resulting in a decrease in both the COD and phenol removal efficiencies to 31 and 23%, respectively. By decreasing the phenol loading rate back to 0.01 gPh^.^L^–1.^d^–1^ (2.2 mgPh^.^gVSS^–1.^d^–1^), the COD and phenol removal efficiencies were gradually recovered during phase III.

### Volatile Fatty Acids (VFAs) Spectrum

**FIGURE 3:**
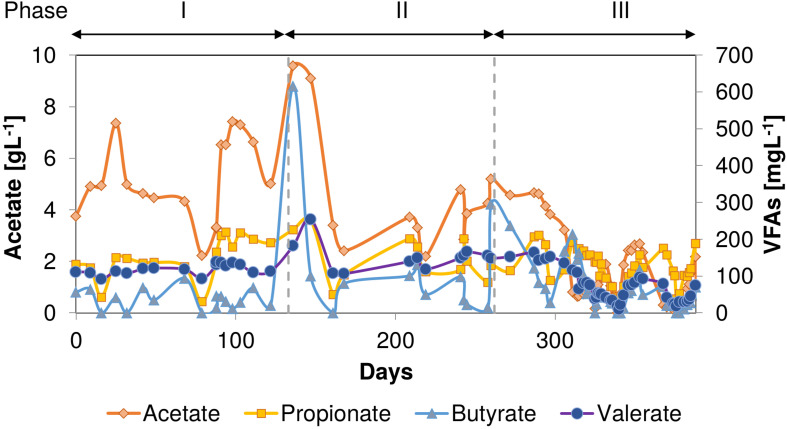
VFAs concentrations during the AnMBR long-term operation at 55°C and 18 gNa^+.^L^–1^. Acetate (left y-axis), Propionate, Butyrate, and Valerate (right y-axis). The decreased of VFAs concentration values on day 79 is due to a stop of feeding.

Throughout the entire thermophilic operation, VFAs were detected in the AnMBR permeate (Figure 3), indicating a limiting methanogenic conversion capacity. The effluent COD mainly consisted of acetate (0.02–9.6 g^.^L^–1^), which peaked at almost 9.6 g^.^L^–1^ between 133 and 144 days when phenol accumulation occurred. Concomitantly, the butyrate concentration increased to 616 mg^.^L^–1^ in this period. In phase II, high concentrations of acetate (5.2 g^.^L^–1^) and to a lesser extent butyrate (295 mg^.^L^–1^) were found at day 260 when phenol again accumulated after an increase in the phenol loading rate. Propionate was most of the time present in the reactor effluent with an average concentration of 129 ± 57 mg^.^L^–1^. The valerate concentration increased to 254 and 156 mg^.^L^–1^ when the reactor performance deteriorated in phases I and II, respectively. In phase III, on day 350, an increase in all VFAs was observed when reactor phenol concentration increased to 124 mgPh^.^L^–1^.

### Conversion Rates and Methanogenic Activity

#### Specific Methanogenic Activity and Phenol Conversion Rates

SMA tests using acetate as the substrate showed a drop in the methanogenic activity of the phenol-degrading biomass in phase I, meanwhile, an increase in the specific phenol conversion rates was observed (Table 2). During phase II the lowest observed SMA was 0.04 ± 0.02 gCOD-CH_4_^.^gVSS^–1^^.^d^–1^, following phenol accumulation at the end of phase I. In phase III when the influent phenol concentration was decreased to 0.2 gPh^.^L^–1^, the SMA of the phenol-degrading biomass increased to 0.13 gCOD-CH_4_^.^gVSS^–1^^.^d^–1^, which was similar to the value observed at the beginning of phase I.

**TABLE 2:** SMA (acetate as the substrate) and phenol conversion rates at different phases of the AnMBR operation under thermophilic conditions.

Phase	Days	SMA [gCOD-CH_4_^.^gVSS^–1.^d^–1^]	AnMBR phenol conversion rate [mgPh^.^gVSS^–1.^d^–1^]
I	0–12	0.13 ± 0.05	1.3 ± 0.6
	43–69	0.08 ± 0.05	8.4 ± 0.3
	100–133	N.D*	21.3 ± 0.2
II	156–198	0.04 ± 0.02	5.9 ± 0.3
	220–241	N.D*	16.0 ± 0.6
	242–262	N.D*	16.9 ± 0.6
III	326–332	0.09 ± 0.07	2.6 ± 0.1
	333–349	N.D	7.6 ± 1.6
	350–388	0.13 ± 0.10	6.3 ± 0.2

*N.D, not determined.

Table 2 also shows the calculated maximum in-reactor phenol conversion rates in the different phases. At the end of phase I, the phenol conversion rate had increased from an initial value of 1.3 to 21.3 ± 0.2 mgPh^.^gVSS^–1.^d^–1^. During the recovery periods of perturbation, i.e., days 156–198, the phenol conversion rate was 5.9 ± 0.3 mgPh^.^gVSS^–1.^d^–1^. From days 220 to 262, the phenol loading rate increased until an average of 16.9 ± 0.6 mgPh^.^gVSS^–1.^d^–1^. In phase III, the phenol conversion rate decreased to the range 2.6 ± 0.1–7.6 ± 1.6 mgPh^.^gVSS^–1.^d^–1^.

#### *Ex situ* Phenol Conversion Rate After Reactor Perturbation

After reactor perturbation at the end of phase II, the phenol conversion rate was assessed in a batch test. Three different feedings were applied with different initial phenol concentrations (see Table 3). After the first feed, the phenol conversion rate was calculated as 4.0 ± 1.4 mgPh^.^gVSS^–1.^d^–1^ after 7 days of incubation. The phenol conversion rate increased to 9.6 ± 2.6 and 10.5 ± 3.3 mgPh^.^gVSS^–1.^d^–1^ after the second and third consecutive feeding respectively.

**TABLE 3:** Phenol conversion rate during three consecutive feedings in the batch test.

Feed	Phenol conversion rate [mgPh^.^gVSS^–1.^d^–1^]	Initial phenol concentration [mgPh^.^L^–1^]	Initial COD concentration (phenol+acetate) [gCOD^.^L^–1^]
1^*st*^	4.0 ± 1.4	109 ± 12	5.1 ± 0.3
2^*nd*^	9.6 ± 2.6	60 ± 3	3.4 ± 0.0
3^*rd*^	10.5 ± 3.3	89 ± 25	3.6 ± 0.1

#### Specific Methanogenic Activity and Phenol Conversion Rates at the End of the Long-Term Operation

Since low acetate-fed SMA values at the end of the long-term thermophilic operation period were observed of 0.13 ± 0.10 gCOD-CH_4_^.^gVSS^–1.^d^–1^, SMA tests were performed with both acetate and hydrogen as electron donor. Likewise, the phenol conversion rate was measured in batch test with an initial phenol concentration of 40 and 60 mgPh^.^L^–1^ (Table 4). The acetate-fed SMA obtained was 0.15 ± 0.04 gCOD-CH_4_^.^gVSS^–1^^.^d^–1^ while the hydrogenotrophic methanogenic activity was 0.34 ± 0.08 gCOD-CH_4_^.^gVSS^–1^^.^d^–1^. A maximum phenol conversion rate of 32.8 ± 0.5 mgPh^.^gVSS^–1.^d^–1^ was found, applying an initial phenol concentration of 60 mgPh^.^L^–1^.

**TABLE 4:** Acetate-fed and hydrogen-fed specific methanogenic activities, as well as phenol conversion rates assessed at two different initial phenol concentrations.

	SMA [gCOD-CH_4_^.^gVSS^–1.^d^–1^]	Phenol conversion rates [mgPh^.^gVSS^–1.^d^–1^]	
		[40 mgPh^.^L**^–^**^1^]_*initial*_	[60 mgPh^.^L**^–^**^1^]_*initial*_
Acetate	0.15 ± 0.04	29.2 ± 0.1	32.8 ± 0.5
H_2_/CO_2_	0.34 ± 0.08		

### Microbial Community Structure Analysis

**FIGURE 4:**
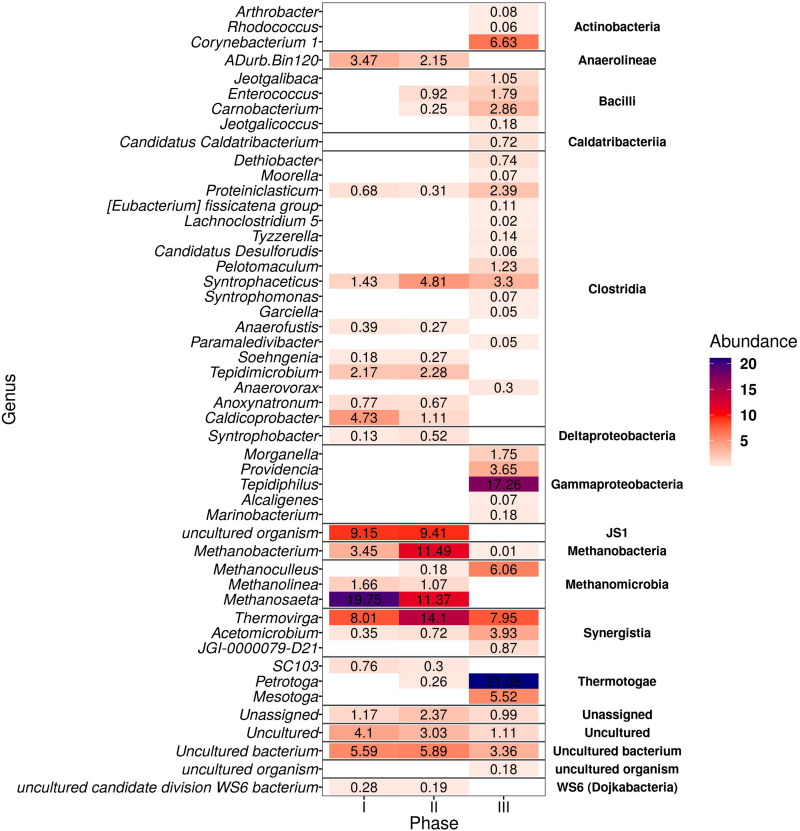
Heatmap of the genera from bacteria and archaea domains in the thermophilic AnMBR that were positive after differential abundance analysis (DESeq2) among the three phases of reactor operation. The color scale ranges from 0 to 22% relative abundance.

The microbial community analysis revealed a total of 141 ASVs with differential abundance across samples. Figure 4 shows the genera from both bacteria and archaea domains with main population changes in relative abundance at the different phases of the thermophilic AnMBR at 18 gNa^+^L^–1^. *Petrotoga* (Thermotogae class) was enriched along with the long-term operation up to 21.1% in phase III. *Thermovirga* (Synergisitia class) increased from 8.0 to 14.1% from phase I to II and then decreased to 7.95% relative abundance in phase III. *Acetomicrobium* also belonging to phylum *Synergistetes* (see Supplementary Figure 1) increased from 0.35% during phase I to 3.93% during phase III. A 100% similarity was found with the specie *Acetomicrobium hydrogeniformans* sp. (see Supplementary Table 1). *Syntrophobacter* affiliated to the class Deltaproteobacteria increased from 0.1 to 0.5% in relative abundance from phase I to phase II; however it was not detected in phase III. A hit of 100% similarity was found for the halophilic bacteria *Syntrophobacter sulfatireducens* sp. Similarly, the relative abundance of WS6 bacterium decreased during the long-term operation. In the case of the Clostridia class, the major abundance decrease was observed with the genera *Caldicoprobacter and Tepidimicrobium*, whereas an increase was observed for *Syntrophaceticus* (3.3%), *Pelotomaculum* (1.2%), and *Proteiniclasticum* (2.4%). Also, *Corynebacterium* and *Enterococcus* genera belonging to Actinobacteria and Bacilli class increased significantly to 6.6 and 1.8%, respectively, in phase III.

*Methanosaeta* was about 19.7% in relative abundance in phase I decreasing to 11.4% in phase II, while it almost disappeared in phase III. The halotolerant *Methanosaeta harundinacea* sp. obtained a 100% similarity on this genus. Concomitantly, *Methanobacterium* increased from 3.5% in phase I to 11.5% in phase II, but significantly dropped in phase III. In phase III, the hydrogenotrophic methanogens *Methanoculleus* increased to 6.1% in relative abundance becoming the dominant archaea while *Methanolinea* decreased significantly.

Figure 4 also illustrates that the most prominent amplicon sequence variants belong to 15 and 2 different classes in the bacterial and archaeal domain, respectively.

For the bacteria microbial community, the abundance of microorganisms of the class Actinobacteria, Bacilli, Synergistia, Gammaproteobacteria, and Thermotogae increased correspondingly to 6.8, 5.9, 22.9, 12.6, and 26.6% at the end of the long-term thermophilic operation.

## Discussion

### Thermophilic AnMBR Performance and Volatile Fatty Acids Spectrum

The COD removal efficiency started to decrease at the end of the applied phenol loading rate of 0.09 gPh^.^L^–1.^d^–1^ (Figure 2B), i.e., before day 96 in phase I, and subsequently acetate concentration increased (Figure 3). The observed deterioration possibly can be ascribed to an accumulation of a phenol conversion intermediate, such as benzoate (Wang and Barlaz, 1998; Tay et al., 2001), impacting acetoclastic methanogens or syntrophic acetate oxidizers. After the phenol loading rate was further increased to 0.12 gPh^.^L^–1.^d^–1^, equivalent to a sludge phenol loading rate of 22.6 mgPh^.^gVSS^–1.^d^–1^, the OLR was decreased to 2.3 gCOD^.^L^–1.^d^–1^ to avoid high volatile fatty acids concentrations in the reactor. The increase in phenol loading rate at the end of phase I, severely impacted COD and phenol conversion, resulting in removal efficiencies below 10%. The phenol concentration in the reactor broth reached 738 mgPh^.^L^–1^ (Figure 2A), which apparently inhibited both phenol conversion and methanogenesis. Similarly, Madigou et al. (2016) reported inhibition of phenol conversion at a concentration of 600 mgPh^.^L^–1^, and biogas production nearly stopped when phenol reached 895 mgPh^.^L^–1^. In order to reduce the phenol concentration in the AnMBR, the phenol loading rate was decreased to 0.02 gPh^.^L^–1.^d^–1^ (3.8 mgPh^.^gVSS^–1.^d^–1^) on day 133 to prevent further intoxication, while the OLR was reduced to 2.0 gCOD^.^L^–1.^d^–1^. In phase II, the reactor performance again deteriorated. Consequently, biogas production almost ceased on day 260 when the reactor phenol concentration reached 616 mgPh^.^L^–1^, inhibiting the methanogenic consortium. Surprisingly in phase III, by applying a phenol loading rate of 0.04 gPh^.^L^–1.^d^–1^ (9.3 mgPh^.^gVSS^–1.^d^–1^) on day 352, phenol and COD removal efficiencies decreased to 50 and 80%, respectively. The latter indicates an increased sensitivity of the biomass to phenol compared to phase I.

The observed maximum phenol conversion rate of 21.3 ± 0.2 mgPh^.^gVSS^–1.^d^–1^ in the AnMBR at the phenol loading rate of 0.12 gPh^.^L^–1.^d^–1^ (22.6 mgPh^.^gVSS^–1.^d^–1^) at 55°C and 18 gNa^+.^L^–1^ was substantially higher than the observed phenol conversion rate of 1.7 mgPh^.^gVSS^–1.^d^–1^ at a loading rate of 0.02 gPh^.^L^–1.^d^–1^ (3.9 mgPh^.^gVSS^–1.^d^–1^), which was observed in our previous work after shifting an AnMBR operation from 35 to 55°C at a sodium concentration of 16 gNa^+.^L^–1^ (Muñoz Sierra et al., 2018b). However, it is lower than the 81.3 mgPh^.^gVSS^–1.^d^–1^ found by others (Wang et al., 2011). It should be noted that in our present work, the AnMBR was exposed to more extreme conditions, combining high phenol concentrations, high temperature, and high salinity (18 gNa^+.^L^–1^) compared to the previous thermophilic studies (see Table 5). Still, our observed phenol conversion rates can be considered low when compared to the 52.7–489 mgPh^.^gVSS^–1.^d^–1^ that was achieved with thermophilic phenol degrading methanogenic enriched consortia (Chen et al., 2008).

**TABLE 5:** Anaerobic phenol conversion in continuous flow reactor systems operated at different temperatures.

Temperature [°C]	Reactor [Volume L]	Operation time [d]	Substrate	Phenol [mgPh^.^L^–1^]	OLR [gCOD^.^L^–1^^.^d^–1^]	Specific phenol conversion rate [mgPh^.^gVSS^–1^^.^d^–1^]	Removal [%]	References
15–18	EGSB-AF [3.5]	415	Phenol, ethanol, butyrate, propionate and acetate	400–1200	5–10	15.4–88.9 (*ex situ*)	>80 (as COD)	Collins et al., 2005
9.5–15	EGSB-AF [3.5]	673	Phenol, ethanol, butyrate, propionate and acetate	500–1000	1–2 (COD_*Ph*_)	Up to 68, 43–137 (15°C, *ex situ*)	50–98	Scully et al., 2006
26	UASB [2.8]	512	Phenol	1260	6	N.A	33–100	Fang et al., 2004
37	ASBR [5]	281	Phenol/phenol, glucose	120–1200	N.D	11–27	>90	Rosenkranz et al., 2013
37	ASBR [5]	200	Phenol	120–1200	N.D	11–31	>90	Franchi et al., 2020
35–55	AnMBR [6.5]	270	Phenol, acetate (high salinity)	100–500	1.86–4.35	1.2–3.5	40–100	Muñoz Sierra et al., 2018b
55	UASB [2.8]	224	Phenol	630	0.9	N.A	59–99	Fang et al., 2006
55	UASB [1]	303	Phenol, phenolics, CGWW	300–500 (total phenols)	1.5–2.5	30.5–81.3 (*ex situ*)	50–60 (total phenols)	Wang et al., 2011
55	AnMBR [6.5]	388	Phenol, acetate (high salinity)	50–800	2–4	21.3–32.8 (*ex situ*)	≤95	This study

*N.A, not available; N.D, not determined; CGWW, coal gasification wastewater.*

Volatile fatty acid concentrations in the permeate, mainly acetate, indicated a limiting methanogenic conversion capacity for the OLR applied. The high observed acetate concentration reaching 9.6 g^.^L^–1^ (Figure 3) when phenol accumulation occurred, could have caused a secondary inhibitory effect to the anaerobic microorganisms such as propionate-oxidizing bacteria (Van Lier et al., 1993). Despite the peaks of 620 and 300 mg^.^L^–1^ of butyrate during the phenol accumulation events, all VFA concentrations (propionate, butyrate, and valerate) remained at a relatively low level, commonly observed in anaerobic reactors under anaerobic thermophilic conditions (Van Lier et al., 1993). Caproate always remained below detection level throughout the entire thermophilic AnMBR operation. Previous studies inferred that at 55°C, phenol-degrading biomass might degrade phenol via *n*-caproate (Evans and Fuchs, 1988; Fang et al., 2006). On the other hand, Hoyos-Hernandez et al. (2014) have demonstrated that thermophilic phenol degradation is possible via the benzoate conversion route, similar to mesophilic conditions, contrasting these previous studies. Even though benzoate was not determined analytically in the AnMBR, the absence of caproate in any of the VFA analyses strongly suggests that the prevailing phenol conversion pathway was likely via benzoate carboxylation at 55°C. Following the proposed pathway, benzoate is subsequently de-aromatized to form cyclohexane carboxylic acid, which is cleaved to heptanoate, degraded further through β-oxidation to form valerate, propionate, and acetate, or directly to propionate and butyrate, which are further degraded to acetate (Liang and Fang, 2010).

### Conversion Rates and Methanogenic Activity

The assessed SMA values found are similar to those reported elsewhere for phenol degrading biomass under thermophilic conditions, i.e., 0.1 gCOD-CH_4_^.^gVSS^–1.^d^–1^ (Fang et al., 2006).

The phenol conversion rate assessed in *ex situ* batch testsincreased to 10.5 ± 3.3 mgPh^.^gVSS^–1.^d^–1^, inferring a 62% recovery of the conversion capacity after about 17 days of incubation. Interestingly, also Chen et al. (2008) found a maximum phenol conversion rate after 10 to 14 days of incubation. The *ex situ* assessed phenol conversion rate at the end of the batch tests was about 61% of the AnMBR conversion rate before phenol accumulation occurred (see Table 3). Apparently, the phenol-degrading biomass could recover from the phenol shocks after a relatively short recovery period, while being exposed to low phenol concentrations in the reactor (<100 mgPh^.^L^–1^). The observed maximum phenol conversion rate of 32.8 ± 0.5 mgPh^.^gVSS^–1.^d^–1^ in batch test at the end of the experiment is higher than the maximum observed phenol conversion rate in the AnMBR during phase I, which likely can be attributed to long-term adaptation of the biomass to phenol after three phases. Comparing our present results with the different studies summarized in Table 5, which are performed in a broad range of temperatures, the observed maximum phenol conversion rates are comparable to those obtained under both mesophilic (Rosenkranz et al., 2013; Franchi et al., 2020) and psychrophilic (Collins et al., 2005; Scully et al., 2006) conditions, using other anaerobic high-rate reactors configurations. Nonetheless, it should be recalled that our present results were obtained under extreme salinity conditions applying sodium concentrations of 18 gNa^+.^L^–1^.

The acetate-fed SMA obtained at the end of the long-term operation was 0.15 ± 0.04 gCOD-CH_4_^.^gVSS^–1^^.^d^–1^ similar to what was observed at the beginning of phase I and at the end of phase III. Remarkably, the hydrogenotrophic methanogenic activity was a factor 2.3 higher (0.34 ± 0.08 gCOD-CH_4_^.^gVSS^–1^^.^d^–1^), which made us hypothesize that methanogenesis of acetate proceeded via syntrophic acetate oxidation coupled to hydrogenotrophic methanogenesis, rather than aceticlastic methanogenesis. Of interest is the relatively high hydrogenotrophic SMA, which indicates that acetate is possibly syntrophically methanised via oxidation to hydrogen and carbon dioxide. Note that syntrophic acetate oxidation is energetically more favorable at elevated temperature and high acetate concentration and is more often observed as the dominant pathway in a large number of thermophilic anaerobic reactors (van Lier, 1996; Westerholm et al., 2016; Li et al., 2020). Moreover, hydrogenotrophic methanogenesis is commonly observed at elevated salt concentrations (De Vrieze et al., 2016).

Based on our present results, follow-up research in a thermophilic AnMBR should reveal the minimum acetate concentration that is required to enhance phenol conversion and to avoid high VFA concentrations in the permeate. In such research, concentrations in the range of 0.3–1.0 gCOD^.^L^–1^ acetate, and 500–800 mgPh^.^L^–1^ phenol are recommended. Applying similar salinity conditions, we propose an OLR of 2.0–2.2 gCOD^.^L^–1^^.^d^–1^ and phenol loading rates of 15–20 mgPh^.^gVSS^–1^^.^d^–1^ in order to maximize the phenol conversion capacity, without compromising the methanogenic activity.

### Microbial Community Structure

*Petrotoga* was enriched to 21.1% during the AnMBR long-term operation. This bacteria has been enriched under anaerobic thermophilic conditions from an oil reservoir containing mostly halophilic species (Dellagnezze et al., 2016). Similarly, high salinity conditions will enrich for salt-tolerant and halophilic *Thermovirga* and *Clostridium* species (Muñoz Sierra et al., 2018a). *Acetomicrobium hydrogeniformans* sp., which also increased during phase III, is found in oil production water and is capable of producing hydrogen. Some species required NaCl for growth (Cook et al., 2018). In the case of the Clostridia class, the genera found *Syntrophaceticus* are known for their capability of syntrophic acetate oxidation, and *Pelotomaculum* play an important role in the conversion of phenol and benzoate under methanogenic conditions (Chen et al., 2008).

The abundance of bacteria of the classes Actinobacteria, Bacilli, Synergistia, Gammaproteobacteria, and Thermotogae increased in phase III. Microorganisms corresponding to these classes have been found in thermophilic saline environments. Clostridia class remained in a comparable relative abundance along with the phases, but with changes at genus level. Especially, microorganisms that belong to this class have been reported as the most essential fermentative bacteria and syntrophic phenol degraders as *Pelotomaculum* (Chen et al., 2009; Muñoz Sierra et al., 2019).

Our results showed a high relative abundance of uncultured microorganisms in the AnMBR. Bacteria belonging to the class JS1, and the candidate phylum Atribacteria (see Supplementary Figure 1) were dominant during phases I (9.15%) and II (9.41%). Microorganisms belonging to Atribacteria, mostly have been found in deep-sea methane-rich sediments (Carr et al., 2015). Lee et al. (2018) suggested a fermentative role of these microorganisms, capable of using various substrates, and syntrophic acetate oxidation coupled with hydrogen scavenging methanogens. Recently, the first culturable representative strain of this phylum was isolated and it was confirmed that it plays a role in hydrogenogenic fermentative metabolism (Katayama et al., 2019). In our case, the highest species similarity found was *Bacillus alkalitolerans strain T3-209*, with only 83%.

In the archaea domain, the microbial dynamics indicated a clear switch from acetotrophic to hydrogenotrophic methanogens in the class Methanomicrobia. Both the microbial community structure and the observation that at the end of the experiment the biomass hydrogenotrophic methanogenic activity was substantially higher than the acetate-fed methanogenic activity, support our hypothesis that acetate conversion switched from aceticlastic methanogenesis to acetate oxidation coupled to hydrogenotrophic methanogenesis.

### Perspectives and Further Research

The observed AnMBR performance perturbation following phenol accumulation indicated that the cultivated thermophilic phenol-degrading biomass was dependent on the presence of active methanogens. A drop in the hydrogenotrophic methanogenic activity may have led to the observed reduced phenol conversion capacity. It should be noted that the entire experiment was performed under high salinity (18 gNa^+.^L^–1^) conditions. Further research will focus on the role of syntrophic acetate oxidation and phenol degradation intermediates (e.g., benzoate). Our present study showed that highly saline phenolic wastewaters indeed could be treated in a thermophilic AnMBR. However, the achievable phenol conversion capacity was restricted to 20 mgPh^.^gVSS^–1.^d^–1^, determining an applicable phenol loading rate of about 0.12 gPh^.^L^–1.^d^–1^. Although thermophilic operation will bring operational energy benefits when treating high-temperature industrial wastewaters with the target of process water reuse, the phenol conversion capacity of the reactor will be lower than when opting for mesophilic operation.

## Conclusion

Maximum COD and phenol removal efficiencies of about 95% were achieved during the long-term thermophilic AnMBR operation at 18 gNa^+.^L^–1^. However, severe perturbations occurred following relatively small increments in the phenol loading rate from 0.01 to 0.12 gPh^.^L^–1.^d^–1^. Moreover, by exceeding a sludge phenol loading rate of 20 mgPh^.^gVSS^–1^^.^d^–1^ the system immediately responded in phenol build-up to concentrations higher than 600 mgPh^.^L^–1^ leading to significant deterioration of methanogenesis. The observed maximum phenol conversion rates were 21.3 ± 0.2 and 32.8 ± 0.5 mgPh^.^gVSS^–1.^d^–1^ in the AnMBR, and in *ex situ* batch test at the end of the reactor operation, respectively. The absence of caproate in the VFAs spectrum inferred that the phenol conversion pathway was likely via benzoate carboxylation. The assessed hydrogenotrophic SMA was a factor 2.3 higher than the acetate-fed SMA. Correspondingly, microbial population dynamics indicated that hydrogenotrophic methanogens were enriched during the long-term operation and Clostridia class was dominant. Overall, thermophilic AnMBR operation under high salinity seemed to be susceptible to sudden increase in phenol loading rate or phenol shocks, indicating that the specific phenol conversion capacity under the studied conditions was limiting the treatment process.

## Data Availability Statement

The datasets presented in this study can be found in online repositories. The names of the repository/repositories and accession number(s) can be found below: https://www.ebi.ac.uk/ena, PRJEB38467.

## Author Contributions

JM designed the experiment. JM and VG performed the experiments, contributed with the reactor’s maintenance, and carried out analytical methods. JM analyzed the data and wrote the manuscript. DC-G did the Bioinformatics analysis. HS and JvL provided feedback that helped shape the research and analysis, and critically revised the manuscript. All authors have read and approved the final manuscript.

## Conflict of Interest

The authors declare that the research was conducted in the absence of any commercial or financial relationships that could be construed as a potential conflict of interest.

## References

[B1] APHA, (2005). *Standard Methods for the Examination of Water and Wastewater.* Washington, DC: American Public Health Association.

[B2] Bolyen E. Rideout J. R. Dillon M. R. Bokulich N. A. Abnet C. C. Al-Ghalith G. A. (2019). Reproducible, interactive, scalable and extensible microbiome data science using QIIME 2. *Nat. Biotechnol.* 37 852–857. 10.1038/s41587-019-0209-9 31341288 PMC7015180

[B3] Busca G. Berardinelli S. Resini C. Arrighi L. (2008). Technologies for the removal of phenol from fluid streams: a short review of recent developments. *J. Hazard. Mater.* 160 265–288. 10.1016/j.jhazmat.2008.03.045 18455866

[B4] Callahan B. J. McMurdie P. J. Rosen M. J. Han A. W. Johnson A. J. A. Holmes S. P. (2016). DADA2: high-resolution sample inference from Illumina amplicon data. *Nat. Methods* 13:581. 10.1038/nmeth.3869 27214047 PMC4927377

[B5] Carr S. A. Orcutt B. N. Mandernack K. W. Spear J. R. (2015). Abundant Atribacteria in deep marine sediment from the Adélie Basin. Antarctica. *Front. Microbiol.* 6:872. 10.3389/fmicb.2015.00872 26379647 PMC4549626

[B6] Chen C.-L. Wu J.-H. Liu W.-T. (2008). Identification of important microbial populations in the mesophilic and thermophilic phenol-degrading methanogenic consortia. *Water Res.* 42 1963–1976. 10.1016/j.watres.2007.11.037 18234274

[B7] Chen C.-L. Wu J.-H. Tseng I. C. Liang T.-M. Liu W.-T. (2009). Characterization of active microbes in a full-scale anaerobic fluidized bed reactor treating phenolic wastewater. *Microbes Environ.* 24 144–153. 10.1264/jsme2.me09109 21566367

[B8] Coates J. D. Coughlan M. F. Colleran E. (1996). Simple method for the measurement of the hydrogenotrophic methanogenic activity of anaerobic sludges. *J. Microbiol. Methods* 26 237–246. 10.1016/0167-7012(96)00915-3

[B9] Collins G. Foy C. McHugh S. Mahony T. O’Flaherty V. (2005). Anaerobic biological treatment of phenolic wastewater at 15–18°C. *Water Res.* 39 1614–1620. 10.1016/j.watres.2005.01.017 15878034

[B10] Cook L. E. Gang S. S. Ihlan A. Maune M. Tanner R. S. McInerney M. J. (2018). Genome sequence of acetomicrobium hydrogeniformans OS1. *Genome Announc.* 6:e00581-18. 10.1128/genomeA.00581-18 29954899 PMC6025917

[B11] De Vrieze J. Raport L. Roume H. Vilchez-Vargas R. Jáuregui R. Pieper D. H. (2016). The full-scale anaerobic digestion microbiome is represented by specific marker populations. *Water Res.* 104 101–110. 10.1016/j.watres.2016.08.008 27522020

[B12] Dellagnezze B. M. Vasconcellos S. P. D. Melo I. S. D. Santos Neto E. V. D. Oliveira V. M. D. (2016). Evaluation of bacterial diversity recovered from petroleum samples using different physical matrices. *Braz. J. Microbiol.* 47 712–723. 10.1016/j.bjm.2016.04.004 27282730 PMC4927652

[B13] Dereli R. K. Ersahin M. E. Ozgun H. Ozturk I. Jeison D. van der Zee F. (2012). Potentials of anaerobic membrane bioreactors to overcome treatment limitations induced by industrial wastewaters. *Bioresour. Technol.* 122 160–170. 10.1016/j.biortech.2012.05.139 22749827

[B14] Duncan J. Bokhary A. Fatehi P. Kong F. Lin H. Liao B. (2017). Thermophilic membrane bioreactors: a review. *Bioresour. Technol.* 243 (Suppl. C), 1180–1193. 10.1016/j.biortech.2017.07.059 28736143

[B15] Evans W. C. Fuchs G. (1988). Anaerobic degradation of aromatic compounds. *Annu. Rev. Microbiol.* 42 289–317. 3059996 10.1146/annurev.mi.42.100188.001445

[B16] Fang H. H. P. Liang D. W. Zhang T. Liu Y. (2006). Anaerobic treatment of phenol in wastewater under thermophilic condition. *Water Res.* 40 427–434. 16406477 10.1016/j.watres.2005.11.025

[B17] Fang H. H. P. Liu Y. Ke S. Z. Zhang T. (2004). Anaerobic degradation of phenol in wastewater at ambient temperature. *Water Sci. Technol.* 49 95–102. 10.2166/wst.2004.0028 14979543

[B18] Franchi O. Cabrol L. Chamy R. Rosenkranz F. (2020). Correlations between microbial population dynamics, bamA gene abundance and performance of anaerobic sequencing batch reactor (ASBR) treating increasing concentrations of phenol. *J. Biotechnol.* 310 40–48. 10.1016/j.jbiotec.2020.01.010 32001255

[B19] Hoyos-Hernandez C. Hoffmann M. Guenne A. Mazeas L. (2014). Elucidation of the thermophilic phenol biodegradation pathway via benzoate during the anaerobic digestion of municipal solid waste. *Chemosphere* 97 115–119. 10.1016/j.chemosphere.2013.10.045 24238916

[B20] Katayama T. Nobu M. K. Kusada H. Meng X.-Y. Yoshioka H. Kamagata Y. (2019). Membrane-bounded nucleoid discovered in a cultivated bacterium of the candidate phylum ‘Atribacteria’. *bioRxiv* 10.1101/728279

[B21] Katoh K. Standley D. M. (2013). MAFFT multiple sequence alignment software version 7: improvements in performance and usability. *Mol. Biol. Evol.* 30 772–780. 10.1093/molbev/mst010 23329690 PMC3603318

[B22] Lee Y. M. Hwang K. Lee J. I. Kim M. Hwang C. Y. Noh H.-J. (2018). Genomic insight into the predominance of candidate phylum atribacteria JS1 lineage in marine sediments. *Front. Microbiol.* 9:2909. 10.3389/fmicb.2018.02909 30555444 PMC6281690

[B23] Levén L. Schnürer A. (2005). Effects of temperature on biological degradation of phenols, benzoates and phthalates under methanogenic conditions. *Int. Biodeteriorat. Biodegrad.* 55 153–160. 10.1016/j.ibiod.2004.09.004

[B24] Li Q. Liu Y. Yang X. Zhang J. Lu B. Chen R. (2020). Kinetic and thermodynamic effects of temperature on methanogenic degradation of acetate, propionate, butyrate and valerate. *Chem. Eng. J.* 396 125–366. 10.1016/j.cej.2020.125366

[B25] Liang D. Fang H. H. P. (2010). “Anaerobic treatment of phenolic wastewaters,” in *Environmental Anaerobic Technology*, ed. Fang H. H. P., (Singapore: World Scientific), 185–205.

[B26] Lin H. Peng W. Zhang M. Chen J. Hong H. Zhang Y. (2013). A review on anaerobic membrane bioreactors: applications, membrane fouling and future perspectives. *Desalination* 314 169–188. 10.1016/j.desal.2013.01.019

[B27] Love M. I. Huber W. Anders S. (2014). Moderated estimation of fold change and dispersion for RNA-seq data with DESeq2. *Genome Biol.* 15:550. 10.1186/s13059-014-0550-8 PMC4302049 25516281

[B28] Madigou C. Poirier S. Bureau C. Chapleur O. (2016). Acclimation strategy to increase phenol tolerance of an anaerobic microbiota. *Bioresour. Technol.* 216 77–86. 10.1016/j.biortech.2016.05.045 27233100

[B29] McMurdie P. J. Holmes S. (2013). Phyloseq: an r package for reproducible interactive analysis and graphics of microbiome census data. *PLoS One* 8:e61217. 10.1371/journal.pone.0061217 23630581 PMC3632530

[B30] Muñoz Sierra J. D. Lafita C. Gabaldón C. Spanjers H. van Lier J. B. (2017). Trace metals supplementation in anaerobic membrane bioreactors treating highly saline phenolic wastewater. *Bioresour. Technol.* 234 106–114. 10.1016/j.biortech.2017.03.032 28319758

[B31] Muñoz Sierra J. D. Oosterkamp M. J. Wang W. Spanjers H. van Lier J. B. (2018a). Impact of long-term salinity exposure in anaerobic membrane bioreactors treating phenolic wastewater: performance robustness and endured microbial community. *Water Res.* 141 172–184. 10.1016/j.watres.2018.05.006 29783170

[B32] Muñoz Sierra J. D. Wang W. Cerqueda-Garcia D. Oosterkamp M. J. Spanjers H. van Lier J. B. (2018b). Temperature susceptibility of a mesophilic anaerobic membrane bioreactor treating saline phenol-containing wastewater. *Chemosphere* 213 92–102. 10.1016/j.chemosphere.2018.09.023 30216817

[B33] Muñoz Sierra J. D. Oosterkamp M. J. Wang W. Spanjers H. van Lier J. B. (2019). Comparative performance of upflow anaerobic sludge blanket reactor and anaerobic membrane bioreactor treating phenolic wastewater: overcoming high salinity. *Chem. Eng. J.* 366 480–490. 10.1016/j.cej.2019.02.097

[B34] Price M. N. Dehal P. S. Arkin A. P. (2010). FastTree 2 - approximately maximum-likelihood trees for large alignments. *PLoS One* 5:e9490. 10.1371/journal.pone.0009490 20224823 PMC2835736

[B35] Ramakrishnan A. Surampalli R. Y. (2013). Performance and energy economics of mesophilic and thermophilic digestion in anaerobic hybrid reactor treating coal wastewater. *Bioresour. Technol.* 127 9–17. 10.1016/j.biortech.2012.09.071 23138053

[B36] Raza W. Lee J. Raza N. Luo Y. Kim K.-H. Yang J. (2019). Removal of phenolic compounds from industrial waste water based on membrane-based technologies. *Ind. Eng. Chem.* 71 1–18. 10.1016/j.jiec.2018.11.024

[B37] Rognes T. Flouri T. Nichols B. Quince C. Mahé F. (2016). VSEARCH: a versatile open source tool for metagenomics. *PeerJ* 4:e2584. 10.7717/peerj.2584 PMC5075697 27781170

[B38] Rosenkranz F. Cabrol L. Carballa M. Donoso-Bravo A. Cruz L. Ruiz-Filippi G. (2013). Relationship between phenol degradation efficiency and microbial community structure in an anaerobic SBR. *Water Res.* 47 6739–6749. 10.1016/j.watres.2013.09.004 24083853

[B39] Scully C. Collins G. O’Flaherty V. (2006). Anaerobic biological treatment of phenol at 9.5*–*15°C in an expanded granular sludge bed (EGSB)-based bioreactor. *Water Res.* 40 3737–3744. 10.1016/j.watres.2006.08.023 17064753

[B40] Spanjers H. Vanrolleghem P. (2016). “Respirometry,” in *Experimental Methods in Wastewater Treatment*, eds van Loosdrecht M. C. M. Nielsen P. H. Lopez-Vazquez C. M. Brdjanovic D., (London: IWA Publishing).

[B41] Tay J.-H. He Y.-X. Yan Y.-G. (2001). Improved anaerobic degradation of phenol with supplemental glucose. *J. Environ. Eng.* 127 38–45.

[B42] van Lier J. B. (1996). Limitations of thermophilic anaerobic wastewater treatment and the consequences for process design. *Antonie van Leeuwenhoek* 69 1–14. 8678474 10.1007/BF00641606

[B43] van Lier J. B. (2008). High-rate anaerobic wastewater treatment: diversifying from end-of-the-pipe treatment to resource-oriented conversion techniques. *Water Sci. Technol.* 57 1137–1148. 10.2166/wst.2008.040 18469383

[B44] Van Lier J. B. Grolle K. C. Frijters C. T. Stams A. J. Lettinga G. (1993). Effects of acetate, propionate, and butyrate on the thermophilic anaerobic degradation of propionate by methanogenic sludge and defined cultures. *Appl. Environ. Microbiol.* 59 1003–1011. 8476278 10.1128/aem.59.4.1003-1011.1993 PMC202229

[B45] van Lier J. B. van der Zee F. P. Frijters C. T. M. J. Ersahin M. E. (2015). Celebrating 40 years anaerobic sludge bed reactors for industrial wastewater treatment. *Rev. Environ. Sci. Biotechnol.* 14 681–702. 10.1007/s11157-015-9375-5

[B46] Villegas L. G. C. Mashhadi N. Chen M. Mukherjee D. Taylor K. E. Biswas N. (2016). A short review of techniques for phenol removal from wastewater. *Curr. Pollut. Rep.* 2 157–167.

[B47] Wang W. Ma W. Han H. Li H. Yuan M. (2011). Thermophilic anaerobic digestion of Lurgi coal gasification wastewater in a UASB reactor. *Bioresour. Technol.* 102 2441–2447. 10.1016/j.biortech.2010.10.140 21112778

[B48] Wang W. Yang K. Muñoz Sierra J. Zhang X. Yuan S. Hu Z. (2017). Potential impact of methyl isobutyl ketone (MIBK) on phenols degradation in an UASB reactor and its degradation properties. *J. Hazard. Mater.* 333 73–79. 10.1016/j.jhazmat.2017.03.033 28342357

[B49] Wang Y.-S. Barlaz M. A. (1998). Anaerobic biodegradability of alkylbenzenes and phenol by landfill derived microorganisms. *FEMS Microbiol. Ecol.* 25 405–418.

[B50] Westerholm M. Moestedt J. Schnürer A. (2016). Biogas production through syntrophic acetate oxidation and deliberate operating strategies for improved digester performance. *Appl. Energy* 179 124–135. 10.1016/j.apenergy.2016.06.061

