# Supplementary material for: Anaerobic Conversion of Saline Phenol-Containing Wastewater Under Thermophilic Conditions in a Membrane Bioreactor
Source: Front Bioeng Biotechnol. 2020 Sep 30;8:565311. doi: 10.3389/fbioe.2020.565311 (PMC7556282; doi:10.3389/fbioe.2020.565311)
Supplement: Supplementary file 1 [file Data_Sheet_1.docx]

Supplementary Material

# Supplementary Figures


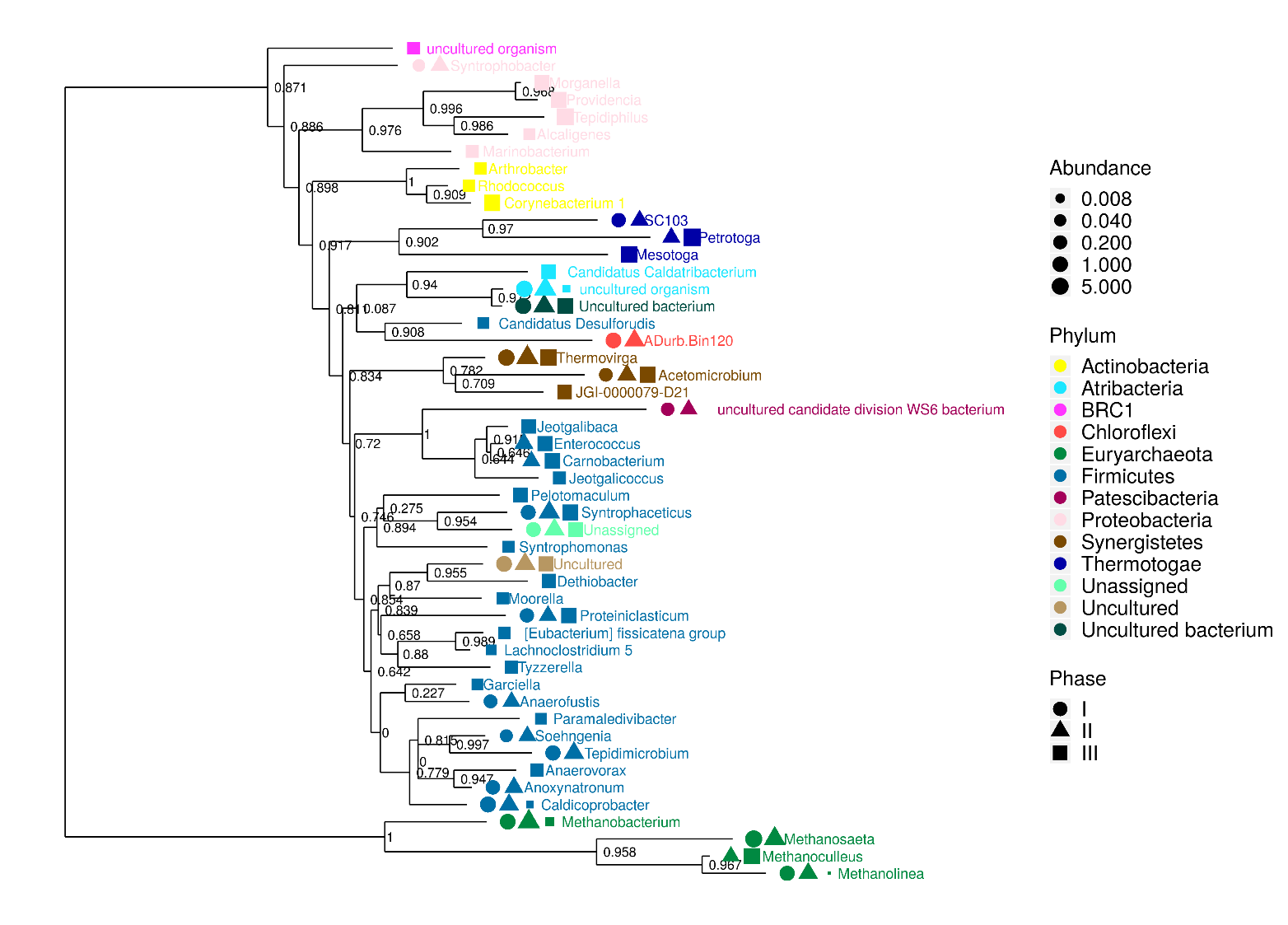


**Supplementary Figure 1.** Phylogenetic tree after differential abundance analysis (DESeq2), indicating the relative abundance of bacteria and archaea genera belonging to their respective phylum at phases I (circle), II (triangle) and III (square) during the long-term thermophilic AnMBR operation. The size of circle, triangle, and square represent the relative abundance, which is presented in Log2 scale (0–5) to facilitate the comparison among phases.

# Supplementary Tables

**Supplementary Table 1.** ASVs with differential abundances within the operational phases and their closest related species hits based on similarity. RA: relative abundance

| **RA.**  **Phase I** | **RA Phase II** | **RA**  **Phase III** | **Phylum** | **Class** | **Genus** | **NCBI_ID** | **Similarity** | **e-value** | **Specie_NCBI** |
| --- | --- | --- | --- | --- | --- | --- | --- | --- | --- |
| 0,0000 | 0,0000 | 0,1068 | Firmicutes | Clostridia | *[Eubacterium] fissicatena group* | NR_144617.1 | 99 | 0 | *Muricomes intestini strain 2PG-424-CC-1* |
| 0,0000 | 0,1655 | 2,0233 | Synergistetes | Synergistia | *Acetomicrobium* | NR_116842.1 | **99,256** | 0 | *Acetomicrobium hydrogeniformans ATCC BAA-1850 strain OS1* |
| 0,3498 | 0,5498 | 1,9090 | Synergistetes | Synergistia | *Acetomicrobium* | NR_116842.1 | **100** | 0 | *Acetomicrobium hydrogeniformans ATCC BAA-1850 strain OS1* |
| 0,8234 | 0,6739 | 0,0000 | Chloroflexi | Anaerolineae | *ADurb.Bin120* | NR_041354.1 | 91,771 | 4,97E-155 | *Bellilinea caldifistulae strain GOMI-1* |
| 2,3608 | 1,2651 | 0,0000 | Chloroflexi | Anaerolineae | *ADurb.Bin120* | NR_041355.1 | 90,524 | 1,08E-146 | *Longilinea arvoryzae strain KOME-1* |
| 0,2842 | 0,2128 | 0,0000 | Chloroflexi | Anaerolineae | *ADurb.Bin120* | NR_040972.1 | 92,25 | 2,3E-158 | *Levilinea saccharolytica strain KIBI-1* |
| 0,0000 | 0,0000 | 0,0689 | Proteobacteria | Gammaproteobacteria | *Alcaligenes* | NR_114959.1 | **100** | 0 | *Alcaligenes aquatilis strain LMG 22996* |
| 0,3935 | 0,2719 | 0,0000 | Firmicutes | Clostridia | *Anaerofustis* | NR_027562.1 | 93,052 | 2,29E-163 | *Anaerofustis stercorihominis strain WAL 14563* |
| 0,0000 | 0,0000 | 0,3002 | Firmicutes | Clostridia | *Anaerovorax* | NR_159296.1 | 97,015 | 0 | *Aminipila butyrica strain FH042* |
| 0,3206 | 0,3488 | 0,0000 | Firmicutes | Clostridia | *Anoxynatronum* | NR_043664.1 | **99,501** | 0 | *Tindallia texcoconensis strain IMP-300* |
| 0,4518 | 0,3192 | 0,0000 | Firmicutes | Clostridia | *Anoxynatronum* | NR_043664.1 | 98,504 | 0 | *Tindallia texcoconensis strain IMP-300* |
| 0,0000 | 0,0000 | 0,0840 | Actinobacteria | Actinobacteria | *Arthrobacter* | NR_116375.1 | **100** | 0 | *Arthrobacter halodurans strain JSM 078085* |
| 4,7289 | 1,1055 | 0,0045 | Firmicutes | Clostridia | *Caldicoprobacter* | NR_117466.1 | 90,347 | 3,93E-146 | *Caldicoprobacter algeriensis strain TH7C1* |
| 0,0000 | 0,0000 | 0,3320 | Atribacteria | Caldatribacteriia | *Candidatus Caldatribacterium* | NR_133741.1 | 82,984 | 1,24E-101 | *Enterococcus xiangfangensis strain 11097* |
| 0,0000 | 0,0000 | 0,1035 | Atribacteria | Caldatribacteriia | *Candidatus Caldatribacterium* | NR_126262.1 | 83,178 | 2,66E-103 | *Melghirimyces profundicolus strain SCSIO 11153* |
| 0,0000 | 0,0000 | 0,2854 | Atribacteria | Caldatribacteriia | *Candidatus Caldatribacterium* | NR_133741.1 | 82,984 | 1,24E-101 | *Enterococcus xiangfangensis strain 11097* |
| 0,0000 | 0,0000 | 0,0565 | Firmicutes | Clostridia | *Candidatus Desulforudis* | NR_159908.1 | 86,385 | 4,29E-126 | *Desulfothermobacter acidiphilus strain 3408-1* |
| 0,0000 | 0,0000 | 0,2118 | Firmicutes | Bacilli | *Carnobacterium* | NR_040926.1 | **99,529** | 0 | *Carnobacterium mobile strain DSM 4848* |
| 0,0000 | 0,0000 | 0,7538 | Firmicutes | Bacilli | *Carnobacterium* | NR_042093.1 | **99,529** | 0 | *Carnobacterium gallinarum strain DSM 4847* |
| 0,0000 | 0,2483 | 1,8979 | Firmicutes | Bacilli | *Carnobacterium* | NR_040926.1 | **99,765** | 0 | *Carnobacterium mobile strain DSM 4848* |
| 0,0000 | 0,0000 | 1,2733 | Actinobacteria | Actinobacteria | *Corynebacterium 1* | NR_074663.1 | **99,267** | 0 | *Corynebacterium glutamicum strain ATCC 13032* |
| 0,0000 | 0,0000 | 4,4771 | Actinobacteria | Actinobacteria | *Corynebacterium 1* | NR_074663.1 | **99,755** | 0 | *Corynebacterium glutamicum strain ATCC 13032* |
| 0,0000 | 0,0000 | 0,8771 | Actinobacteria | Actinobacteria | *Corynebacterium 1* | NR_074663.1 | **99,511** | 0 | *Corynebacterium glutamicum strain ATCC 13032* |
| 0,0000 | 0,0000 | 0,7376 | Firmicutes | Clostridia | *Dethiobacter* | NR_117747.2 | 89,189 | 3,05E-137 | *Desulfofundulus thermocisternus DSM 10259* |
| 0,0000 | 0,1360 | 0,3037 | Firmicutes | Bacilli | *Enterococcus* | NR_117976.1 | **99,765** | 0 | *Enterococcus viikkiensis strain IE3.2* |
| 0,0000 | 0,7862 | 1,4868 | Firmicutes | Bacilli | *Enterococcus* | NR_114453.1 | **100** | 0 | *Enterococcus malodoratus strain ATCC 43197* |
| 0,0000 | 0,0000 | 0,0454 | Firmicutes | Clostridia | *Garciella* | NR_144613.1 | 95,782 | 0 | *Irregularibacter muris strain 2PG-426-CC-4.2* |
| 0,0000 | 0,0000 | 0,9466 | Firmicutes | Bacilli | *Jeotgalibaca* | NR_125553.1 | **100** | 0 | *Jeotgalibaca dankookensis strain EX-07* |
| 0,0000 | 0,0000 | 0,0988 | Firmicutes | Bacilli | *Jeotgalibaca* | NR_156899.1 | 98,118 | 0 | *Jeotgalibaca arthritidis strain 1805-02* |
| 0,0000 | 0,0000 | 0,1834 | Firmicutes | Bacilli | *Jeotgalicoccus* | NR_025644.1 | **100** | 0 | *Jeotgalicoccus psychrophilus strain YKJ-115* |
| 0,0000 | 0,0000 | 0,3133 | Synergistetes | Synergistia | *JGI-0000079-D21* | NR_116842.1 | 89,951 | 2,36E-143 | *Acetomicrobium hydrogeniformans ATCC BAA-1850 strain OS1* |
| 0,0000 | 0,0000 | 0,5543 | Synergistetes | Synergistia | *JGI-0000079-D21* | NR_116842.1 | 90,196 | 5,08E-145 | *Acetomicrobium hydrogeniformans ATCC BAA-1850 strain OS1* |
| 0,0000 | 0,0000 | 0,0240 | Firmicutes | Clostridia | *Lachnoclostridium 5* | NR_112174.1 | **99,75** | 0 | *[Clostridium] sphenoides JCM 1415 strain ATCC 19403* |
| 0,0000 | 0,0000 | 0,1835 | Proteobacteria | Gammaproteobacteria | *Marinobacterium* | NR_114163.1 | **99,059** | 0 | *Marinobacterium georgiense strain NBRC 102606* |
| 0,0000 | 0,0000 | 5,4197 | Thermotogae | Thermotogae | *Mesotoga* | NR_117646.2 | **100** | 0 | *Mesotoga infera strain VNs100* |
| 0,0000 | 0,0000 | 0,0998 | Thermotogae | Thermotogae | *Mesotoga* | NR_117755.1 | **99,766** | 0 | *Mesotoga prima MesG1.Ag.4.2 strain mesG1.Ag.4.2* |
| 0,2915 | 1,0345 | 0,0016 | Euryarchaeota | Methanobacteria | *Methanobacterium* | NR_042895.1 | **100** | 0 | *Methanobacterium aarhusense strain H2-LR* |
| 3,1623 | 10,4576 | 0,0107 | Euryarchaeota | Methanobacteria | *Methanobacterium* | NR_028247.1 | **99,74** | 0 | *Methanobacterium subterraneum strain A8p* |
| 0,0000 | 0,1833 | 5,3983 | Euryarchaeota | Methanomicrobia | *Methanoculleus* | NR_043961.1 | **100** | 0 | *Methanoculleus receptaculi strain ZC-2* |
| 0,0000 | 0,0000 | 0,6619 | Euryarchaeota | Methanomicrobia | *Methanoculleus* | NR_116881.1 | **99,737** | 0 | *Methanoculleus hydrogenitrophicus strain HC* |
| 1,6613 | 1,0700 | 0,0020 | Euryarchaeota | Methanomicrobia | *Methanolinea* | NR_028163.1 | 96,842 | 5,87E-179 | *Methanolinea tarda strain NOBI-1* |
| 0,3862 | 0,3370 | 0,0000 | Euryarchaeota | Methanomicrobia | *Methanosaeta* | NR_043203.1 | **100** | 0 | *Methanosaeta harundinacea strain 8Ac* |
| 18,7628 | 10,6408 | 0,0000 | Euryarchaeota | Methanomicrobia | *Methanosaeta* | NR_043203.1 | 98,177 | 0 | *Methanosaeta harundinacea strain 8Ac* |
| 0,5975 | 0,3961 | 0,0000 | Euryarchaeota | Methanomicrobia | *Methanosaeta* | NR_043203.1 | 98,698 | 0 | *Methanosaeta harundinacea strain 8Ac* |
| 0,0000 | 0,0000 | 0,0721 | Firmicutes | Clostridia | *Moorella* | NR_113196.1 | 94,104 | 0 | *Moorella thermoacetica strain JCM 9319* |
| 0,0000 | 0,0000 | 0,1804 | Proteobacteria | Gammaproteobacteria | *Morganella* | NR_043751.1 | **99,529** | 0 | *Morganella morganii subsp. sibonii strain DSM 14850* |
| 0,0000 | 0,0000 | 0,1738 | Proteobacteria | Gammaproteobacteria | *Morganella* | NR_043751.1 | **99,765** | 0 | *Morganella morganii subsp. sibonii strain DSM 14850* |
| 0,0000 | 0,0000 | 1,2535 | Proteobacteria | Gammaproteobacteria | *Morganella* | NR_043751.1 | **99,294** | 0 | *Morganella morganii subsp. sibonii strain DSM 14850* |
| 0,0000 | 0,0000 | 0,1406 | Proteobacteria | Gammaproteobacteria | *Morganella* | NR_043751.1 | **99,765** | 0 | *Morganella morganii subsp. sibonii strain DSM 14850* |
| 0,0000 | 0,0000 | 0,0516 | Firmicutes | Clostridia | *Paramaledivibacter* | NR_152683.1 | 92,5 | 4,93E-160 | *Wukongibacter baidiensis strain DY30321* |
| 0,0000 | 0,0000 | 1,2320 | Firmicutes | Clostridia | *Pelotomaculum* | NR_041320.1 | 96,02 | 0 | *Pelotomaculum isophthalicicum JI* |
| 0,0000 | 0,1833 | 20,9057 | Thermotogae | Thermotogae | *Petrotoga* | NR_169443.1 | **99,762** | 0 | *Petrotoga japonica strain AR80* |
| 0,0000 | 0,0769 | 0,1771 | Thermotogae | Thermotogae | *Petrotoga* | NR_169443.1 | **99,286** | 0 | *Petrotoga japonica strain AR80* |
| 0,0000 | 0,0000 | 2,3907 | Firmicutes | Clostridia | *Proteiniclasticum* | NR_118108.1 | 96,25 | 0 | *Youngiibacter fragilis 232.1* |
| 0,6849 | 0,3074 | 0,0000 | Firmicutes | Clostridia | *Proteiniclasticum* | NR_115875.1 | 99 | 0 | *Proteiniclasticum ruminis DSM 24773 strain D3RC-2* |
| 0,0000 | 0,0000 | 1,2339 | Proteobacteria | Gammaproteobacteria | *Providencia* | NR_115880.1 | **99,765** | 0 | *Providencia rettgeri strain NCTC 11801* |
| 0,0000 | 0,0000 | 1,2361 | Proteobacteria | Gammaproteobacteria | *Providencia* | NR_104913.1 | **100** | 0 | *Providencia sneebia DSM 19967 strain A* |
| 0,0000 | 0,0000 | 1,1780 | Proteobacteria | Gammaproteobacteria | *Providencia* | NR_115880.1 | **99,529** | 0 | *Providencia rettgeri strain NCTC 11801* |
| 0,0000 | 0,0000 | 0,0564 | Actinobacteria | Actinobacteria | *Rhodococcus* | NR_104776.1 | **100** | 0 | *Nocardia coeliaca strain DSM 44595* |
| 0,7578 | 0,2956 | 0,0000 | Thermotogae | Thermotogae | *SC103* | NR_025969.1 | 80 | 1,64E-80 | *Pseudothermotoga subterranea strain SL1* |
| 0,1822 | 0,2660 | 0,0000 | Firmicutes | Clostridia | *Soehngenia* | NR_117382.1 | 100 | 0 | *Soehngenia saccharolytica strain DSM 12858* |
| 1,4282 | 4,8061 | 3,2960 | Firmicutes | Clostridia | *Syntrophaceticus* | NR_116297.1 | 97,022 | 0 | *Syntrophaceticus schinkii strain Sp3* |
| 0,1312 | 0,5202 | 0,0000 | Proteobacteria | Deltaproteobacteria | *Syntrophobacter* | NR_043073.1 | **100** | 0 | *Syntrophobacter sulfatireducens strain TB8106* |
| 0,0000 | 0,0000 | 0,0291 | Firmicutes | Clostridia | *Syntrophomonas* | NR_122058.1 | 93,706 | 6,63E-179 | *Syntrophomonas wolfei strain Goettingen G311* |
| 0,0000 | 0,0000 | 0,0436 | Firmicutes | Clostridia | *Syntrophomonas* | NR_122058.1 | 93,473 | 3,08E-177 | *Syntrophomonas wolfei strain Goettingen G311* |
| 0,9400 | 0,8926 | 0,0000 | Firmicutes | Clostridia | *Tepidimicrobium* | NR_117380.1 | **99,749** | 0 | *Tepidimicrobium ferriphilum strain DSM 16624* |
| 0,4299 | 0,3961 | 0,0000 | Firmicutes | Clostridia | *Tepidimicrobium* | NR_116042.1 | 97,243 | 0 | *Tepidimicrobium xylanilyticum strain PML14* |
| 0,2623 | 0,1833 | 0,0000 | Firmicutes | Clostridia | *Tepidimicrobium* | NR_117379.1 | 97 | 0 | *[Clostridium] ultunense DSM 10521* |
| 0,5392 | 0,8040 | 0,0000 | Firmicutes | Clostridia | *Tepidimicrobium* | NR_117379.1 | 96,75 | 0 | *[Clostridium] ultunense DSM 10521* |
| 0,0000 | 0,0000 | 4,2899 | Proteobacteria | Gammaproteobacteria | *Tepidiphilus* | NR_025556.1 | **99,059** | 0 | *Tepidiphilus margaritifer strain N2-214* |
| 0,0000 | 0,0000 | 12,9670 | Proteobacteria | Gammaproteobacteria | *Tepidiphilus* | NR_025556.1 | **99,294** | 0 | *Tepidiphilus margaritifer strain N2-214* |
| 3,7598 | 5,8938 | 4,5585 | Synergistetes | Synergistia | *Thermovirga* | NR_074606.1 | 93,3 | 1,06E-166 | *Thermovirga lienii strain DSM 17291* |
| 0,1676 | 0,3192 | 0,0000 | Synergistetes | Synergistia | *Thermovirga* | NR_074606.1 | 93,564 | 2,28E-168 | *Thermovirga lienii strain DSM 17291* |
| 0,6704 | 1,2887 | 0,0000 | Synergistetes | Synergistia | *Thermovirga* | NR_074606.1 | 93,812 | 4,9E-170 | *Thermovirga lienii strain DSM 17291* |
| 0,2623 | 0,3488 | 0,0000 | Synergistetes | Synergistia | *Thermovirga* | NR_074606.1 | 93,052 | 4,94E-165 | *Thermovirga lienii strain DSM 17291* |
| 0,0000 | 0,0000 | 0,5665 | Synergistetes | Synergistia | *Thermovirga* | NR_074606.1 | 92,804 | 2,3E-163 | *Thermovirga lienii strain DSM 17291* |
| 2,5794 | 5,3913 | 0,0341 | Synergistetes | Synergistia | *Thermovirga* | NR_074606.1 | 93,564 | 2,28E-168 | *Thermovirga lienii strain DSM 17291* |
| 0,3498 | 0,7449 | 0,0000 | Synergistetes | Synergistia | *Thermovirga* | NR_074606.1 | 93,086 | 4,94E-165 | *Thermovirga lienii strain DSM 17291* |
| 0,0000 | 0,0000 | 1,0825 | Synergistetes | Synergistia | *Thermovirga* | NR_074606.1 | 92,804 | 2,3E-163 | *Thermovirga lienii strain DSM 17291* |
| 0,2186 | 0,1182 | 0,0000 | Synergistetes | Synergistia | *Thermovirga* | NR_074606.1 | 91,872 | 1,08E-156 | *Thermovirga lienii strain DSM 17291* |
| 0,0000 | 0,0000 | 1,7043 | Synergistetes | Synergistia | *Thermovirga* | NR_074606.1 | 93,548 | 2,28E-168 | *Thermovirga lienii strain DSM 17291* |
| 0,0000 | 0,0000 | 0,1410 | Firmicutes | Clostridia | *Tyzzerella* | NR_113408.1 | **99,504** | 0 | *Anaerotignum propionicum strain JCM 1430* |
| 0,0000 | 0,0000 | 0,0404 | Bacteroidetes | Bacteroidia | *Unassigned* | NR_042987.1 | 92,857 | 3,97E-171 | *Petrimonas sulfuriphila strain BN3* |
| 0,0000 | 0,0000 | 0,3402 | BRC1 | Unassigned | *Unassigned* | NR_104861.1 | 82,009 | 2,7E-93 | *Caldanaerobacter subterraneus strain DSM 13054* |
| 0,0000 | 0,0000 | 0,0280 | Firmicutes | Clostridia | *Unassigned* | NR_102767.2 | 97,882 | 0 | *Syntrophothermus lipocalidus strain DSM 12680* |
| 0,0000 | 0,0000 | 0,0666 | Firmicutes | Clostridia | *Unassigned* | NR_126221.1 | 88,759 | 6,96E-144 | *Desulfuribacillus alkaliarsenatis strain AHT28* |
| 0,0000 | 0,0000 | 0,0709 | Firmicutes | Clostridia | *Unassigned* | NR_108634.1 | 89,176 | 1,94E-144 | *Moorella humiferrea strain 64-FGQ* |
| 0,1530 | 0,1655 | 0,0000 | Patescibacteria | Microgenomatia | *Unassigned* | NR_156873.1 | 78,345 | 2,05E-64 | *Actinophytocola xanthii strain KCTC 39690* |
| 0,2186 | 1,8444 | 0,0145 | Firmicutes | Clostridia | *Unassigned* | NR_114349.1 | 85,981 | 9,29E-123 | *Calderihabitans maritimus strain KKC1* |
| 0,0000 | 0,0000 | 0,1157 | Planctomycetes | Phycisphaerae | *Unassigned* | NR_164615.1 | 81,638 | 9,21E-88 | *Sedimentisphaera salicampi strain ST-PulAB-D4* |
| 0,2113 | 0,1182 | 0,0000 | Acidobacteria | c5LKS83 | *Unassigned* | NR_152720.1 | 80,976 | 4,36E-81 | *Marinibaculum pumilum strain H2* |
| 0,0000 | 0,0000 | 0,1319 | Proteobacteria | Gammaproteobacteria | *Unassigned* | NR_025510.1 | 99,294 | 0 | *Alicycliphilus denitrificans K601* |
| 0,0000 | 0,0000 | 0,0214 | Firmicutes | Clostridia | *Unassigned* | NR_119284.1 | 96,526 | 0 | *Pseudoclostridium thermosuccinogenes strain DSM 5807* |
| 0,5902 | 0,2424 | 0,0000 | Thermotogae | Thermotogae | *Unassigned* | NR_044583.2 | 90,676 | 2,46E-158 | *Kosmotoga olearia TBF 19.5.1* |
| 0,0000 | 0,0000 | 0,1575 | Firmicutes | Clostridia | *Unassigned* | NR_041236.1 | 89,082 | 1,1E-136 | *Lutispora thermophila DSM 19022 strain EBR46* |
| 3,1551 | 2,4415 | 0,0000 | Firmicutes | Clostridia | *uncultured* | NR_044205.1 | 88,586 | 6,61E-134 | *Dethiobacter alkaliphilus AHT 1* |
| 0,2113 | 0,2246 | 0,0000 | Chloroflexi | Anaerolineae | *uncultured* | NR_109544.1 | 89,526 | 1,41E-140 | *Ornatilinea apprima strain P3M-1* |
| 0,0000 | 0,0000 | 0,0402 | Firmicutes | Clostridia | *uncultured* | NR_125623.1 | 88,806 | 1,42E-135 | *Proteinivorax tanatarense strain Z-910* |
| 0,2405 | 0,2069 | 0,0000 | Chloroflexi | Anaerolineae | *uncultured* | NR_109544.1 | 89,027 | 3,06E-137 | *Ornatilinea apprima strain P3M-1* |
| 0,4955 | 0,1537 | 0,0000 | Firmicutes | Clostridia | *uncultured* | NR_125623.1 | 88,308 | 3,08E-132 | *Proteinivorax tanatarense strain Z-910* |
| 0,0000 | 0,0000 | 1,0733 | Deferribacteres | Deferribacteres | *uncultured* | NR_158118.1 | 93,677 | 6,63E-179 | *Petrothermobacter organivorans strain ANA* |
| 4,9840 | 4,4455 | 0,0000 | Atribacteria | JS1 | *uncultured bacterium* | NR_163643.1 | 82,904 | 4,45E-101 | *Bacillus alkalitolerans strain T3-209* |
| 0,1530 | 0,1537 | 0,0000 | Tenericutes | Mollicutes | *uncultured bacterium* | NR_042955.1 | 83,411 | 2,65E-103 | *Acholeplasma brassicae 0502* |
| 0,1312 | 0,8099 | 0,0000 | Firmicutes | Clostridia | *uncultured bacterium* | NR_117466.1 | 90,819 | 1,81E-149 | *Caldicoprobacter algeriensis strain TH7C1* |
| 0,1239 | 0,3251 | 0,0000 | Firmicutes | Clostridia | *uncultured bacterium* | NR_117466.1 | 87,561 | 2,46E-128 | *Caldicoprobacter algeriensis strain TH7C1* |
| 0,1967 | 0,1596 | 0,0000 | Aegiribacteria | uncultured bacterium | *uncultured bacterium* | NR_159236.1 | 83,565 | 1,59E-105 | *Desulfonatronum parangueonense strain PAR180* |
| 0,0000 | 0,0000 | 0,0205 | Proteobacteria | Deltaproteobacteria | *uncultured bacterium* | NR_025746.1 | 86,946 | 1,19E-131 | *Desulfofaba fastidiosa strain P2* |
| 0,0000 | 0,0000 | 2,6964 | Bacteroidetes | Bacteroidia | *uncultured bacterium* | NR_156071.1 | 80,471 | 2,71E-83 | *Labilibacter aurantiacus strain HQYD1* |
| 0,0000 | 0,0000 | 0,5281 | Chloroflexi | Anaerolineae | *uncultured bacterium* | NR_117865.1 | 88,424 | 8,6E-133 | *Thermanaerothrix daxensis strain GNS-1* |
| 0,0000 | 0,0000 | 0,1148 | Actinobacteria | Coriobacteriia | *uncultured bacterium* | NR_146815.1 | 89,926 | 2,34E-143 | *Olegusella massiliensis strain KHD7* |
| 0,2769 | 0,1892 | 0,0000 | Patescibacteria | WS6 (Dojkabacteria) | *uncultured candidate*  *division WS6 bacterium* | NR_044490.1 | 79,851 | 5,61E-75 | *Butyricicoccus pullicaecorum strain 25-3* |
| 6,0478 | 5,5805 | 0,0046 | Atribacteria | JS1 | *uncultured organism* | NR_163643.1 | 82,904 | 4,45E-101 | *Bacillus alkalitolerans strain T3-209* |
| 3,1041 | 3,8248 | 0,0000 | Atribacteria | JS1 | *uncultured organism* | NR_163643.1 | 83,138 | 9,56E-103 | *Bacillus alkalitolerans strain T3-209* |
| 0,0000 | 0,0000 | 0,1807 | BRC1 | uncultured organism | *uncultured organism* | NR_136805.1 | 84,434 | 5,68E-110 | *Marinithermofilum abyssi strain SCSIO 11157* |
|  |  |  |  |  |  |  |  |  |  |
